# Application and Optimization of the Rheological Model for a Hydrophobically Associating Dendrimer Polymer

**DOI:** 10.3390/polym14091747

**Published:** 2022-04-26

**Authors:** Shijie Zhu, Xinsheng Xue, Jian Zhang, Shilun Zhang, Zhezhi Liu

**Affiliations:** 1 State Key Laboratory of Offshore Oil Exploitation, Beijing 100028, China; xuexsh@cnooc.com.cn (X.X.); zhangjian@cnooc.com.cn (J.Z.); zhangshl53@cnooc.com.cn (S.Z.); 2 Institute of Petroleum and Natural Gas Engineering, Chongqing University of Science & Technology, Chongqing 401331, China; 2019062@cqust.edu.cn; 3 China National Offshore Oil Corporation (CNOOC) Research Institute Ltd., Beijing 100028, China

**Keywords:** dendrimer hydrophobically associating polymer, viscoelasticity, rheology, constitutive equation, characteristic relaxation time

## Abstract

Polymer flooding is one of the most important enhancing oil recovery (EOR) technologies in the world. With the optimization of polymer synthesis, the performance of polymer solutions has been greatly improved, which can adapt to more complex oil and gas reservoirs. However, with the continuous improvement of the properties of polymer solutions, the elastic property of polymer solutions is significantly improved, and the rheological law has also changed. This series of changes affects the application of polymer flooding reservoir numerical simulation technology. Therefore, constructing an accurate description model and precise limitation conditions is particularly important. The rheological curve with a wide shear range (0.1~10,000 s^−1^) and the viscoelasticity of the two polymers (partially hydrolysed polyacrylamide (HPAM) and dendritic hydrophobic association polymer (DHAP)) were analyzed and tested by a rotating rheometer. The results showed that under the experimental conditions, the rheological curve of both polymers can be described by the Carreau rheological model. Meanwhile, the structural viscosity of the hydrophobically associating polymer solution (DHAP) greatly improved the elasticity of the solution and led to the change of elastic modulus. Considering the influence of elastic characteristics on the rheological curve, the relaxation time spectrum derived from small vibration experimental data was used to limit the characteristic relaxation time, that is, the value range of λ. It was observed that the experimental data were highly matched with the nonlinear regression fitting curve of the Carreau rheological model. Therefore, the relationship between different test parameters should be fully considered while studying the rheological constitutive equation of viscoelastic fluid, so as to optimize and improve the equation of it.

## 1. Introduction

In China, more than 90% of the oilfields are developed by water injection, and an increasing number of oilfields have entered the high water cut stage (including reservoirs with various complex conditions). The application of polymer flooding-based enhanced oil recovery (EOR) technology is imminent [1,2]. Polymer flooding technology realizes mobility control by increasing the viscosity of the displacement phase solution and reducing the seepage capacity of the displacement phase, so as to improve the sweep efficiency and displacement efficiency in the process of displacement, and effectively enhances oil recovery. The most commonly used polymer is partially hydrolysed polyacrylamide, but with the water injection development of more complex oil and gas reservoirs, partially hydrolysed polyacrylamide cannot easily meet the needs of mobility control [3,4]. However, complex reservoir conditions necessitate higher requirements for the performance of polymer solution applications, thus promoting the rapid development of polymer synthesis technology [5]. Currently, the synthesis of polymer flooding agents mainly focuses on improving the performance of the solution by chemically modifying the molecular structure of the polymer and introducing functional groups to produce functional polymers [6,7,8]. More recent studies focus on the synthesis and characterization of the hydrophobic groups linked to polymers of dendritic structures [9]. Based on the synthesis of dendritic polymers, a large number of researchers have conducted comparative performance studies, and the results are shown in Table 1.

Different scholars have compared and analysed various polymers under different research conditions and found that dendritic polymers have more solution properties than other polymers (partially hydrolysed polyacrylamide, comb polymers), and they have a stronger viscosity increasing ability, shear resistance and temperature resistance.

**Figure 1:**
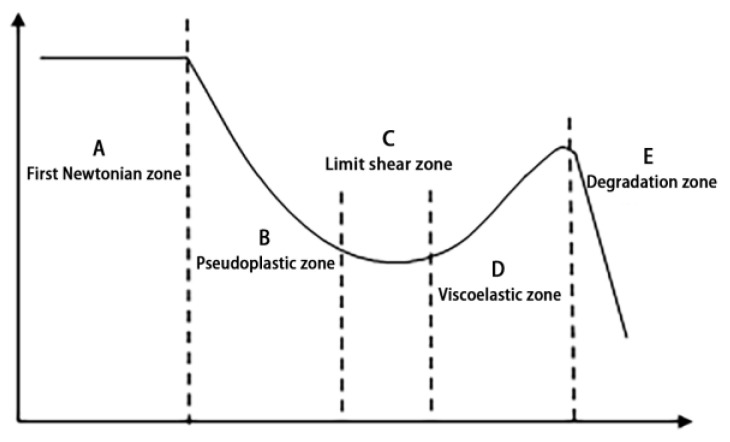
The complete rheological curve of the polymer solution (*x* axis—shear rate; *y* axis—shear viscosity).

Typically, the understanding of the polymer rheological curve has three main stages, including the first Newton section, false plastic section and the second Newton section. However, the effect of the complex structural characteristics on the polymer solution’s performance is also more complex. More and more scholars recognize that the complete rheological curve should be characterized by zero-shear, pseudoplastic, limit-shear, viscoelastic, and degradation sections [14]: (1) The false plastic section is the most common “shear thinning” characteristic section of a polymer for oil displacement, as shown in Figure 1-(B) [15]. (2) The zero-shear section is shown in Figure 1-(A). At this time, the measured viscosity is zero-shear viscosity. Under very small shear stress, as in Newtonian fluids, the solution viscosity remains high [16]. (3) The limit shear section (C) occurs for the following reasons: Once the shear rate increases to a certain extent, the molecular orientation reaches the limit state, and the orientation no longer changes with the shear rate. The polymer solution complies with the Newtonian flow law, and the apparent viscosity becomes a constant, namely, the limit Newtonian section. (4) Although viscoelastic and degradation zones are rare in conventional studies, the relevant literature confirms their existence [17].

A polymer solution will encounter strong shear in the process of field application, resulting in its ultimate shear section. Therefore, it is very important for calculation and application to clarify the polymer rheological formula after characterizing the limit shear section. In summary, the mathematical model of the zero-shear viscosity section, shear thinning section and extreme shear section can be effectively characterized, and the Carreau Yasuda model is the most complete model for describing the rheology of polymers for oil displacement [18,19,20] (see Formula (1)).

(1) $$\mu =\left(\left(\mu_{0}-\mu_{inf}\right){\left[1+{\left(\lambda r\right)}^{a}\right]}^{\frac{n-1}{a}}+\mu_{inf}\right)$$

where *μ* is the apparent viscosity, mPa·s; *r* is the shear rate, s^−1^; *μ*_0_ is the zero-shear viscosity, mPa·s; *μ_inf_* is the limit shear viscosity, mPa·s; *λ* is the characteristic relaxation time, s; *a* is the Carreau constant; and *n* is the Carreau exponent.

At present, there are obvious problems with the fitting application of the constitutive equation: the minimum square sum of the error between the modelled data and the actual data is too large; there are many unknown parameters (*n*, *μ_0_*, *a*); the characteristic relaxation time fluctuates greatly in the fitting result parameters; and it is difficult to identify the elastic characteristics of the viscoelastic fluid, especially in the rheological curve, when fitting the concentration of different polymer solutions with low accuracy regarding the characteristic parameter value of the solution. Therefore, it is necessary to establish a model to produce an accurate numerical simulation.

This paper obtains the rheology and viscoelasticity of dendritic polymer solution (DHAP) through indoor experiments [9,21]. Using the Carreau–Yasuda model, our goal is to apply the viscoelastic modulus experimental data, establish the characteristic relaxation time constraints of the polymer solution, conduct nonlinear regression of the polymer rheological curves of different concentrations, and establish the characteristic parameters with a regular gradient to ensure the accuracy of the fitting.

## 2. Experiment and Theoretical Foundations

### 2.1. Experimental Conditions

**Figure 2:**
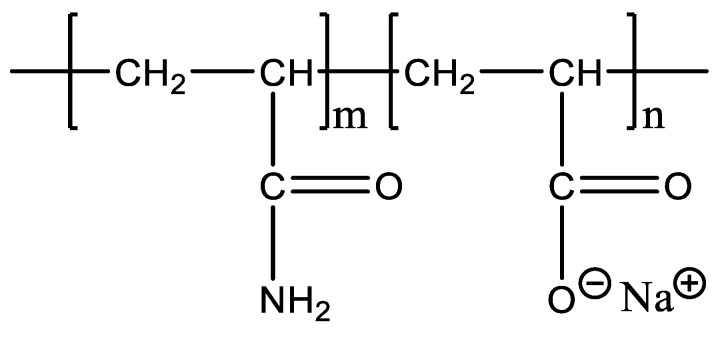
The molecular formula of HPAM.

**Figure 3:**
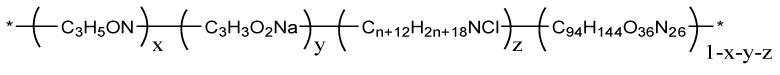
The molecular formula of DHAP.

Polymers used for the experiment: partially hydrolysed polyacrylamide (HPAM) [22], with a relative molecular weight of 20,000 kg/mol, hydrolysis degree of 26.3%, and solid content of 88.9% (Refining and Chemical Company, Daqing, China), as shown in Figure 2; and dendrimer hydrophobically associating polymer (DHAP), with a relative molecular weight of 6000 kg/mol, hydrolysis degree of 25%, hydrophobic group content of 0.6 mol%, and branching degree of 1, the molecular formula of which is shown in Figure 3 (State Key Laboratory of Southwest Petroleum University independent synthesis) [21].

Experimental saline: 3000 mg/L sodium chloride, simulating the effect of cations on the polymer solution.

Experimental instruments: HAAKE RheoStress 600 rheometer (Thermo Electron (Karlsruhe) GmbH, Karlsruhe, Germany); mechanical mixer (Aika (Guangzhou) instrument equipment Co., Ltd., Guangzhou, china); 1000 mL volumetric bottle (Chengdu Kelong Chemical Reagent Factory, Chengdu, China); electronic balance with 0.01% precision (Chengdu Kelong Chemical Reagent Factory, Chengdu, China); and dry petri dish (Chengdu Kelong Chemical Reagent Factory, Chengdu, China).

Experimental temperature: 20 °C.

Rheological determination of the solution [23]: HPAM and DHAP solutions with concentrations of 1000 mg/L, 1400 mg/L, 2000 mg/L and 2500 mg/L were prepared separately. An RS600 rheometer was used to determine the rheological characteristics of the polymer solutions. The CR rotation step was adopted with a shear rate set from $\dot{ɣ}$ = 0.01 s^−1^ to $\dot{ɣ}$ = 10,000 s^−1^, while the dual barrel test system and dg41ti rotor were used in the test.

Determination of the viscoelasticity of the solution [24]: We prepared polymer HPAM and DHAP solutions with solution concentrations of 1000 mg/L, 1400 mg/L, 2000 mg/L and 2500 mg/L. An RS600 rheometer was used to determine the viscoelastic characteristics of the polymer solutions, with angular frequencies from 10 rad/s to 0.01 rad/s and step lengths of 4.

### 2.2. Mathematical Analysis Theory

#### 2.2.1. Calculation of the Relaxation Time Spectrum

The relaxation time spectrum is the most general function for describing the dependence of material viscoelasticity on time or frequency, as well as for describing the full properties of the fluid manifest and the contribution of the sum of all the modes of motion where the relaxation time varies [25]. Test data of the dissipation modulus and a typical linear viscoelastic equation (Maxwell model) were used to analyse linear viscoelastic interval data. The storage modulus and dissipation modulus of the polymer melt can be expressed in discrete form. As shown in Equations (3) and (4), the discrete relaxation time spectrum of the material can be calculated (*λi*, *gi*) [26,27].

(2) $$G^{\prime }\left(\omega \right)=\sum_{i=1}^{N}g_{i}\frac{{\left(\omega \lambda_{i}\right)}^{2}}{1+{\left(\omega \lambda_{i}\right)}^{2}}$$

(3) $$G^{\prime \prime }\left(\omega \right)=\sum_{i=1}^{N}g_{i}\frac{\omega \lambda_{i}}{1+{\left(\omega \lambda_{i}\right)}^{2}}$$

where *ω* is the shear oscillation frequency, *N* is the number of Maxwell motion units, *N* Group (*λ_i_*, *g_i_*) is the discrete relaxation time spectrum constituting the material, *λ_i_* is the relaxation time, and *g_i_* is the relaxation modulus.

#### 2.2.2. Calculation of the Dynamic Modulus

The dynamic modulus can be obtained according to the polymer *G′*, *G″*; see Formula (4). The dynamic modulus represents the different response characteristics of the material under different external load actions. Due to the lag of the phase difference, the stress is divided into two parts: the elastic contribution, which is linear to the strain rate, and the viscous contribution, which is linear with the strain rate.

(4) $$G^{*}=G^{\prime }+zG^{\prime \prime }$$

where G* is the dynamic modulus, Pa; *G′* is the elastic modulus, Pa; *G″* is the viscosity modulus, Pa; and *z* is the phase angle, °.

The calculation formula for the first normal stress difference is shown in Formula (5) [28]:

(5) $$N_{1}=2G^{\prime }{\left[1+{\left(\frac{G^{\prime }}{G^{\prime \prime }}\right)}^{2}\right]}^{0.7}\;$$

where *N*_1_ is the first normal stress difference, Pa; *G′* is the elastic modulus, Pa; and *G″* is the viscosity modulus, Pa.

We refer to the expression of the dynamic modulus in reference [29] as Formula (6).

(6) $$G^{*}\left(\omega \right)=G^{\prime }+\sum_{i=1}^{N}\frac{i\left(\omega \lambda_{i}\right)G^{\prime \prime }}{1+\left(\omega \lambda_{i}\right)}$$

The relaxation modulus represents the relaxation performance of the material, which is the ratio of the material stress to strain at certain temperatures and stress conditions. Formula (7) is established using a generalized Maxwell model for the relaxation modulus.

(7) $$g\left(i\right)=G^{\prime }+\sum_{i=1}^{N}G^{\prime \prime }e^{-t/\lambda_{i}}$$

#### 2.2.3. Nonlinear Regression Viscosity Curve

The viscosity experimental data were fitted into the Carreau–Yasuda model (see Formula (1)) with MATLAB software to obtain the characteristic parameters of rheology.

## 3. Results and Discussion

### 3.1. Viscoelasticity Properties of the Polymer Solution for Oil Displacement

#### 3.1.1. Dynamic Oscillating Experiment

**Figure 4:**
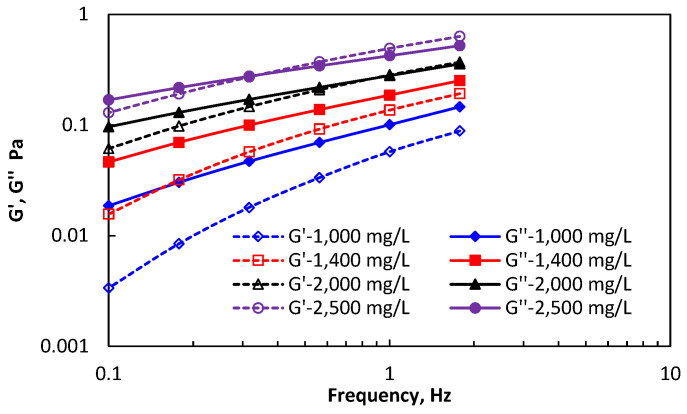
The elastic and viscous modulus of the HPAM.

**Figure 5:**
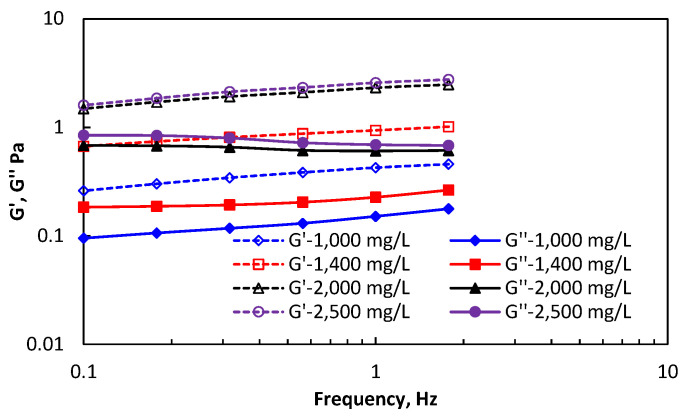
The viscous and elastic moduli of DHAP at different concentrations.

The linear viscoelastic interval of HPAM and DHAP solutions are both between 0.1 Hz~1.7 Hz. The elastic and viscous modulus of the two kinds of polymers in the linear viscoelastic intervals are shown in Figure 4 and Figure 5.

The energy storage/dissipation modulus of HPAM varies at different concentrations. The dissipation modulus (*G*″) at concentrations of 1000 mg/L and 1400 mg/L is dominant in the entire oscillation frequency range, showing strong viscous characteristics. After the concentration increases to 2000 mg/L, the storage modulus increased more in the low-frequency oscillation range, and gradually dominated when the concentration was 2500 mg/L, which indicates that polymer HPAM shows elastic properties only at a high concentration and oscillation frequency. The viscoelasticity of HPAM is mainly determined by the viscosity characteristics, including its flow in porous media. If it does not have strong elastic deformation characteristics, the whole flow resistance comes from viscosity.

DHAP exhibits an absolutely dominant elastic modulus, which is much greater than the viscosity modulus, at four solution concentrations. Before the critical association concentration, the presence of a bendy structure enhanced the spatial arrangement of the hydrophobic group on the branch chain, which somewhat enhanced the molecular beam structure, thus increasing the deformation resistance and showing a large elastic modulus. After the critical joint concentration, the “cluster” spatial collective behaviour was formed, and the deformation resistance was greatly improved.

By improving the molecular structure features and increasing the intermolecular action force, the elastic solution performance of polymer DHAP is stronger.

**Figure 6:**
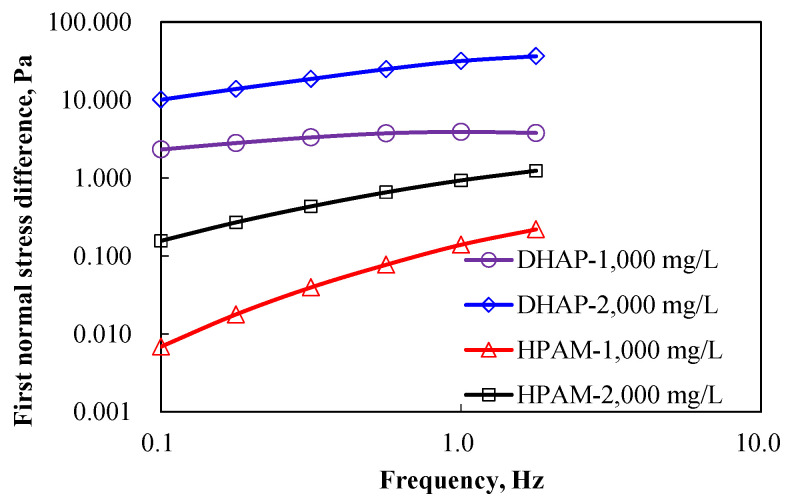
The first normal stress difference of DHAP and HPAM at different concentrations.

Two polymer experimental datasets with solution concentrations of 1000 mg/L and 2000 mg/L were selected for the first normal stress difference calculation (see Formula (5)) and calculated in Figure 6.

The first normal stress difference of polymer DHAP is shown in Figure 6. The value of the first normal stress difference improves significantly compared to the HPAM. Both the bough space and hydrophobic joint do not increase significantly before the critical joint concentration, which shows that the structural hydrophobic connected polymer has strong elastic modulus characteristics, which significantly enhance the deformation resistance ability of the solution, in contrast to HPAM.

#### 3.1.2. The Characteristic Parameter-Limited Optimization of the Relaxation Time

(1) The dynamic modulus data (see Formula (5)) for the polymers HPAM and DHAP are shown in Table 2.

As shown in Table 2, the dynamic moduli of the polymers HPAM and DHAP show an upward trend with increasing solution concentration and oscillation frequency. According to its fitted power-law formula, the G* variation ranges of the two HPAM and DHAP polymer solutions within the studied concentration range are 0.01~1 Pa and 0.27~2 Pa, respectively. Among these, the polymer DHAP dynamic modulus shows no upward trend regularity compared to HPAM, and the 1000 mg/L curve below the critical contracting concentration (1400 mg/L) is different from the curve above the critical contracting concentration. This is the effect of the binding action of the polymer on its complex number modulus features. The polymer DHAP with a concentration of 2500 mg/L has a complex modulus over 1 Pa at 0.1 Hz, which is stronger than the complex modulus of the HPAM.

(2) The relaxation time spectra of the polymers HPAM and DHAP (see Formulas (2) and (3)) are shown in Table 3.

The relaxation time spectrum of partially hydrolysed polyacrylamide HPAM has relatively obvious regularity, and as the relaxation time increases, the relaxation modulus significantly decreases. As the concentration of the polymer solution increases, similar features are presented. This is determined by the molecular structure characteristics of the linear polymer HPAM. Its aggregation behaviour demonstrates the phenomenon of “particle” accumulation, and the increase in solution concentration increases its aggregation behaviour and the accumulation body becomes larger; however, this does not change the essential intermolecular forces, which result in a more obvious regularity [24]. The relaxation time spectrum of the polymer DHAP is significantly distinguished from that of the polymer HPAM; the regularity of the relaxation modulus no longer significantly decreases with relaxation time, but has a low point at a relaxation time of 0.1 s. The analysis shows that when the relaxation time is 0.01 s, the viscosity modulus remains high; when reaching 0.1 s, the viscosity model decreases without triggering the elastic modulus, so the modulus decreases greatly. Furthermore, with the increase in the relaxation time, the trigger of the elastic function feature increases the plural modulus and results in relatively high modulus characteristics. The relaxation time increases again, and the plural model drops substantially. The polymer DHAP has larger relaxation time mathematical variables than HPAM. The enhanced elastic and viscosity moduli of the contracting action perform well on the relaxation time spectrum.

Through the formula transformation of the relaxation modulus and dynamic modulus, the experimental data can limit the relaxation time range of the dynamic modulus under experimental conditions (Formula (7)), as shown in Table 4.

### 3.2. Rheological Properties of the Polymer Solution for Oil Displacement

#### 3.2.1. Viscosity Curve Characteristics of the Polymer

**Figure 7:**
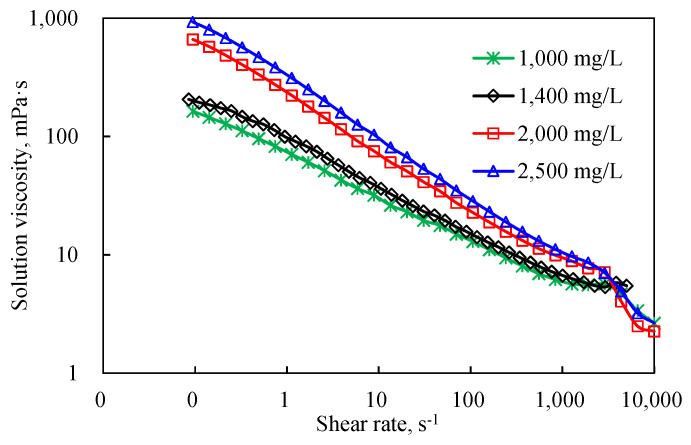
The viscosity curve of the HPAM.

**Figure 8:**
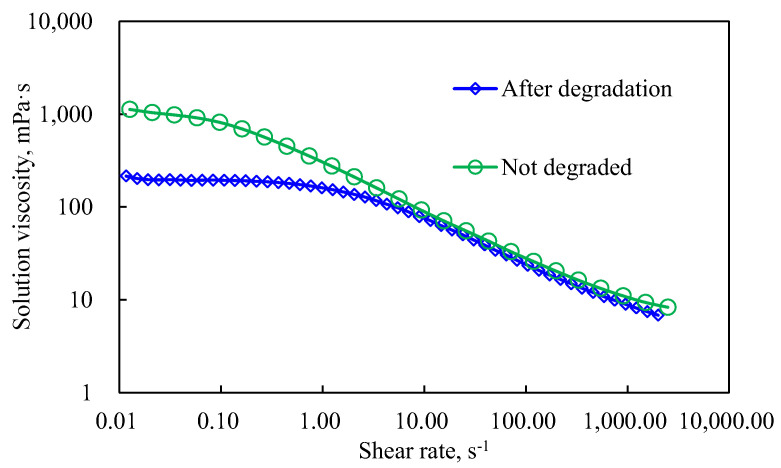
Rheological curve before and after degradation (2500 mg/L).

**Figure 9:**
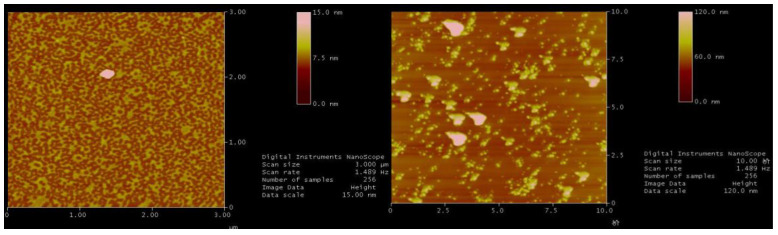
Effect of shearing on microstructure of HPAM [11].

**Figure 10:**
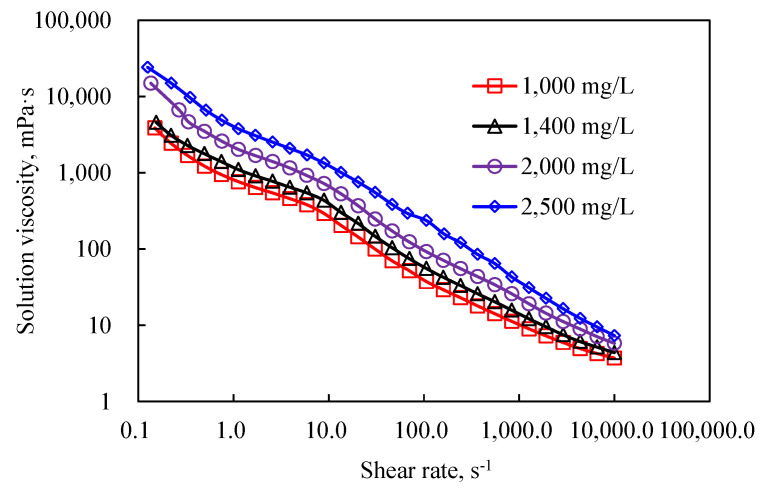
DHAP viscosity curves at different concentrations.

The results of the viscosity curves for HPAM and DHAP are shown in Figure 7, Figure 8, Figure 9 and Figure 10.

As shown in Figure 7, the viscosity curve characteristics change, which indicates that the polymer solution has reached the critical shear rate. Polymers that exceed the critical rate will undergo mechanical degradation. The polymer solution after degradation was retested for the rheological curve test. Compared with the rheological curve of the polymer without degradation (see Figure 8 and Figure 9), the performance of the degraded polymer solution becomes worse, mainly in the obvious decrease in zero-shear viscosity. However, above the critical shear rate, the 2000 mg/L and 2500 mg/L concentrations with higher solution viscosities plummeted under the current conditions; on the other hand, the 1400 mg/L and lower concentrations showed shear thickening intervals and degradation segments. This is because a higher concentration of a polymer solution implies a closer molecular thread group and stronger interaction force. The corresponding external force affects the adjacent keys of the main chain, altering the normal key angle. When the external force is further increased to a value greater than that of the chemical bond, the external force will tear off the molecular chain and directly destroy the molecular structure. Thus, it cannot show the elastic viscosity increasing effect, so it enters the mechanical degradation and viscosity reduction phase. Overall, the polymer solution HPAM, with no mechanical shear within the critical shear rate, still exhibits a typical power-law characteristic of “shear thinning”.

The viscosity curve features of the branchy structure of DHAP are in turn different from those of HPAM and can be described as presenting a distinct “two-section formula” for the whole fluid, showing the viscosity with a low shear rate at one section, and a viscosity rapidly decreasing with shear rate in the other section. The DHAP polymer spatial structure is strong, is affected by the hydrophobic joint effect, and has a strong adhesion capacity and low shear rate on the molecular chain change, so the viscosity decline is slow; with the shear rate, the molecular chain conformation, or entanglement, is opened, and the molecular joint is greatly weakened, finally leading to a rapid decline in the solution viscosity.

#### 3.2.2. Results of the Viscosity Curve Simulation of the Polymer Solution

After imposing the characteristic relaxation time constraints, the fitting calculations are shown in Table 5.

Through the mandatory constraints on the characteristic relaxation time range, the influence of unknown parameters and algorithms on the fitting process is decreased, creating more accurate characteristic relaxation time value conditions and greatly improving the research significance of the fitting data. The rheological curves formed by the above data under the application of the formula are highly consistent with the experimentally measured results. The zero-shear viscosity of the polymer solution increases with increasing concentration.

## 4. Conclusions

1. The DHAP polymer solution is dominated by elastic modulus characteristics and shows a strong first difference, while the HPAM polymer solution is dominated by the viscosity modulus. The increase in the elastic modulus of a polymer solution will directly affect the presentation of the viscous rheological curve, and the rheological model needs to be further modified and limited.
2. The relaxation time spectrum derived by small oscillation experimental data is used to limit the characteristic relaxation time of the polymer solution (value range of λ). Then, the Carreau–Yasuda rheological model is applied to fit the rheology law of the dendrimer hydrophobic-linked polymer solution DHAP with strong elastic action. This not only provides a higher accuracy fit (matching the experimental data), but also avoids the calculation errors caused by nonlinear regression calculations. It provides a basis and help for the optimization of the constitutive equation of polymer solutions in numerical simulation technology.

## Figures and Tables

**Table 1:** Comparison of dendritic polymer solutions with other polymers.

Research Scholar	Research Content
Shijie Zhu [10]	Partially hydrolysed polyacrylamide (HPAM) and dendritic hydrophobic association polymer (DHAP) are compared with independently synthetic dendrimer polymers
Leiting Shi [11]	HAWP is compared with independently synthetic dendrimer polymers
Neha [12]	Influence of excluded volume interactions on the dynamics of dendrimer and star polymers in layered random flow is analysed
Haipeng Xing [13]	The effects of different bendy structures on the performance of the polymer solution are compared

**Table 2:** Dynamic modulus of polymers at different concentrations.

Type	HPAM		DHAP	
Concentration, mg/L	Formulas	Fitting accuracy	Formulas	Fitting accuracy
1000	G* = 0.1152f^0.7606^	R2 = 0.9974	G* = 0.4468f^0.1985^	R2 = 0.9923
1400	G* = 0.2298f^0.6465^	R2 = 0.9955	G* = 0.9689f^0.1414^	R2 = 0.9972
2000	G* = 0.3956f^0.5209^	R2 = 0.9966	G* = 2.3758f^0.1541^	R2 = 0.9945
2500	G* = 0.6459f^0.4677^	R2 = 0.9973	G* = 2.6475f^0.1562^	R2 = 0.9961
Note	G* within the study	0.1~1 Pa	G* within the study	0.27~2 Pa

**Table 3:** The relaxation spectral characteristics of the two polymers.

Type	HPAM				DHAP			
Concentration	1000	1400	2000	2500	1000	1400	2000	2500
*λ_i_*, s	*g*, Pa				*g*, Pa			
0.01	5.12	6.27	3.21	5.04	4.42	6.67	4.30	10.14
0.1	0.18	0.05	0.89	1.28	8.87 × 10^−9^	0.12	1.55	1.06
1	0.10	0.21	0.28	0.46	0.13	0.26	0.60	0.72
10	0.01	0.04	0.13	0.25	0.19	0.30	1.02	1.21
100	5.9 × 10^−10^	1.65 × 10^−9^	4.83 × 10^−9^	1.68 × 10^−9^	0.15 × 10^−7^	0.01 × 10^−6^	5.95 × 10^−6^	5.94 × 10^−6^

**Table 4:** The polymer relaxation time range was studied within the concentration range.

Polymer Type	1000 mg/L	1400 mg/L	2000 mg/L	2500 mg/L
HPAM	λ < 0.01	λ < 0.01	0.01 < λ < 0.1	0.01 <λ < 0.1
DHAP	0.5 < λ < 1	0.5 <λ < 1	1 < λ < 2	1 < λ < 2

**Table 5:** Calculation results of the two polymer solutions (Carreau–Yasuda model).

Polymer	Concentration, mg/L	*μ*_0_	*μ_inf_*	λ	*a*	*n*
HPAM	1000	852.9	4.4	0.002	0.155	−0.250
	2000	2499	4.8	0.005	0.176	−0.306
	2500	7542.9	5.3	0.008	0.152	−0.253
DHAP	1000	3250.5	5.5	0.869	0.420	0.131
	2000	6710	15.4	1.46	0.391	−0.589
	2500	16,890	17.2	1.687	0.443	0.215

## Data Availability

The data supporting the findings of this study are available from the corresponding author upon reasonable request.
